# Between conflict and climate: how drought deepens displacement in war-torn Sudan

**DOI:** 10.1093/inthealth/ihaf144

**Published:** 2025-12-16

**Authors:** Sarah Ali Yahya Adam, Arwa Mohamed, Ibrahim Suleiman, Damilare Akintunde, Iyas Dawood, Rawa Badri

**Affiliations:** Research and Development, End the Neglect Initiative, Khartoum 11111, Sudan; Faculty of Medicine, University of Bahri, Khartoum, Sudan; Sudan Office, Sudanese American Medical Association (SAMA), Port Sudan 31111, Sudan; Research and Development, End the Neglect Initiative, Khartoum 11111, Sudan; Department of Family Medicine, Sudan Medical Specialization Board, Khartoum, Sudan; Research and Development, End the Neglect Initiative, Khartoum 11111, Sudan; Department of Family Medicine, Sudan Medical Specialization Board, Khartoum, Sudan; Research and Development, End the Neglect Initiative, Khartoum 11111, Sudan; Department of Community Medicine, Ahmadu Bello University, Zaria, Nigeria; Research and Development, End the Neglect Initiative, Khartoum 11111, Sudan; Faculty of Medicine, Omdurman Islamic University, Khartoum, Sudan; Research and Development, End the Neglect Initiative, Khartoum 11111, Sudan; Mycetoma Research Centre, University of Khartoum, Khartoum, Sudan

**Keywords:** climate, conflict, displacement, droughts, Sudan

## Abstract

The persistent conflict in Sudan has worsened the impacts of the prolonged droughts, destroying crops and livestock and severely undermining people’s means of survival. Furthermore, the scarcity of water and fertile land has increased competition over resources between communities, further displacing populations. This displacement leads to worsening the crisis, straining resources, spreading disease and overwhelming healthcare. For instance, rainfall, which is one of the most devastating climate changes, can reach up to 427.70 mm in one city. Consequently, Sudan confronts widespread famine threats, while ongoing hostilities significantly restrict humanitarian organizations' ability to provide assistance. Effective solutions must focus on strengthening community resilience through strategic investments, comprehensive emergency preparedness, environmentally sustainable practices and sustained peace-building initiatives that would generate substantial benefits for human welfare and economic development.

## Double crisis—conflict and climate stressors collide

Disasters, especially healthcare crises, are driven by either environmental or human-made factors or both. Currently, Sudan is facing a devastating era in which both conflict and climate influencers combined are causing humanitarian crises, especially healthcare ones.

Sudan is one of the countries located in the Sahel and Horn of Africa, which is most likely to be affected by climate extremes such as frequent droughts, extreme heat waves and spells of heavy rain. Recent climate change has brought alterations in the monsoonal dynamics and desertification process in the country.^1^ Rainfall in Sudan has also become increasingly unpredictable, characterized by late onset, shorter rainy seasons and intense localized storms. This leads to both agricultural disruption and increased flooding risks in urban and rural settings. Northern and central Sudan experience frequent drought cycles that significantly affect crop yields, water availability and pasture productivity, threatening both food security and livelihoods.^2^ Average annual rainfall can reach 427.70 mm in the Western state cities in the country.^1^

Drought is a critical environmental stressor that can intensify underlying social, economic and political tensions, especially in a fragile state like Sudan. In regions where livelihoods depend heavily on rain-fed agriculture and pastoralism, such as Darfur, Kordofan and eastern Sudan, recurrent droughts diminish access to vital natural resources like water and pasture. This lack of resources typically results in farmer–herder conflict over competition for land. Droughts have also been a long-term cause of population displacement, state fragility and grievances among marginalized populations. Internally displaced persons (IDPs) have become a persistent humanitarian challenge. They are forced to move from rural areas affected by drought, violence or resource scarcity to overcrowded camps or fragile urban settlements.^3^ These displacement sites often lack basic infrastructure, adequate housing, clean water and sanitation services, exacerbating public health risks and social tension.

The conflict between the Sudanese Armed Forces (SAF) and the Rapid Support Forces (RSF), which began in April 2023, persisted through 2024, causing severe humanitarian crises across Sudan. By the year’s end, 11.6 million people were internally displaced—the highest globally—with one-half residing in Darfur. Widespread violence, especially in El Fasher, Aj Jazirah and Sennar, forced repeated displacements and limited aid access. More than 24.5 million people faced acute food insecurity, with famine declared in Zamzam camp and other areas. Humanitarian deliveries were disrupted for months, worsening conditions in North Darfur, South Kordofan and surrounding states.^3^

## Humanitarian fallout—fragile systems and escalating needs

According to the 2025 Humanitarian Needs and Response Plan, approximately 20.3 million individuals have been identified as requiring humanitarian health assistance. Among these, 10.2 million are women and girls, 10.6 million are children, 1 million are older adults and 7.4 million are IDPs. Additionally, 1.1 million people are facing catastrophic levels of need, while 12.4 million and 6.6 million are experiencing extreme and severe needs, respectively. In six states—Central Darfur, West Darfur, South Darfur, North Darfur, South Kordofan and Blue Nile—the majority of residents require humanitarian health support. Meanwhile, in 10 states—South Darfur, North Darfur, Khartoum, Al Jazirah, White Nile, North Kordofan, West Kordofan, East Darfur, Gedaref and Sennar—the number of people requiring humanitarian health assistance exceeds 1 million in each. Multiple disease outbreaks, including cholera, dengue, malaria, measles, diphtheria and circulating vaccine-derived poliovirus type 2, are currently affecting several states. Out of the 20 million individuals requiring urgent, life-saving humanitarian health assistance, an estimated 477 000 are pregnant, and within the coming year, approximately 187 127 460 newborns are expected to face complications. An estimated 31 000 to 95 000 pregnant women are expected to experience childbirth complications necessitating a cesarean section as per the World Health Organization (WHO). In addition to the physical impact, 2.1 million children and care-providers are in need of psychological support.^4^ Food insecurity remains a major challenge, driven by ongoing conflict, market failure, limited humanitarian access and mass displacement. At present, 30 out of Sudan’s 189 localities are facing or are at high risk of famine.^5^

In North Darfur, areas like Al Fasher and Zamzam are especially affected, remaining under siege and largely inaccessible to humanitarian aid. The situation worsened significantly following a major attack on Zamzam camp in early April 2025, which caused civilian deaths and forced large numbers of people to flee toward Tawila and Dar As Salam localities in North Darfur. Ongoing insecurity in Al Fasher has led to continued displacement toward Tawila and nearby areas.^5^

The impact of the conflict has extended to the destruction of Sudan’s fragile infrastructure and its vulnerable natural environment. The conflict presents significant threats to biodiversity and environmental stability, with lasting impacts on Sudanese society such as increased climate vulnerability, widespread deforestation and deteriorating soil quality.^6^

## Pathways forward—building resilience amid crises (key recommendations)

### Short-term interventions

Promoting peacebuilding and preventing conflict between the SAF and the RSF in regions such as Kordofan, Darfur and Khartoum will save lives, reduce displacement and enable the return of >9 million IDPs and 3 million refugees. Establishing peace will create a stable environment for recovery and yield economic benefits, with estimated savings of nearly US$5 billion per year.^7^

Sudan should be prioritized for climate justice and adaptation funding, including the ‘Climate Finance for Developing Countries’ and the ‘Loss and Damage Fund’, to address the combined effects of conflict and climate change. Currently, only a small share of these funds reaches low-income countries like Sudan, and only about one-quarter benefits Africa as a whole.^8^

Strengthening and scaling up climate-resilient agricultural practices—such as using drought-resistant crops, rainwater harvesting and solar-powered irrigation—should align with the food and agriculture organization’s Plan of Action for Sudan.^9^ Distribution of drought-resistant seeds should begin in the 30 localities currently facing famine.^5^

### Long-term interventions

Investing in climate-resilient infrastructure, beginning in war-affected areas such as Khartoum, Kordofan and Al Fasher, will yield high returns: up to US$4 for every US$1 invested.^10^ Humanitarian and development agencies should integrate climate risk into their planning, as only a fraction of funds currently target preparedness.^7^

Finally, strengthening early warning systems and empowering civil society organizations (CSOs) and community-based organizations (CBOs) will enhance local governance amid weakened state authority.^7–9^

## Data Availability

No new data were generated or analyzed to support this research.
